# Severe Cyanoacrylate Granuloma Following Cyanoacrylate Closure for Varicose Veins in a Patient with Immune Modulation: A Case Report

**DOI:** 10.3400/avd.cr.25-00144

**Published:** 2026-03-31

**Authors:** Takahiro Imai, Daisuke Harada, Yoichi Mizutani, Soutarou Katsui

**Affiliations:** 1 Department of Vascular Surgery, Nishinokyo Hospital, Nara, Nara, Japan; 2 Sakaimachi Oike Pathology Clinic, Kyoto, Kyoto, Japan; 3 Department of Radiology, Nishinokyo Hospital, Nara, Nara, Japan

**Keywords:** cyanoacrylate closure, cyanoacrylate granuloma, immunomodulatory therapy

## Abstract

We report a case of severe cyanoacrylate granuloma (CAG) that developed shortly after cyanoacrylate closure (CAC) in a patient receiving dupilumab. A 74-year-old female had an uneventful postoperative course until the day before initiation of dupilumab; however, on postoperative day (POD) 16, 1 day after starting dupilumab, she developed fever and painful inguinal nodules. Computed tomography revealed multiple subcutaneous nodules without abscess formation. Despite systemic corticosteroids and antihistamines, the lesions progressed, requiring repeated bilateral excisions through POD 93. This case suggests that immune modulation at the time of cyanoacrylate exposure may influence the severity and progression of granulomatous inflammation after CAC.

## Introduction

Cyanoacrylate closure (CAC) is a widely accepted, minimally invasive treatment for saphenous vein reflux, offering durable vein occlusion without the need for tumescent local anesthesia. While generally safe, rare complications such as phlebitis, hypersensitivity, and cyanoacrylate granuloma (CAG) have been described.^1)^ CAG represents a chronic foreign-body reaction, typically occurring several months after treatment, and its true incidence, risk factors, and optimal management remain incompletely understood. Most reported cases present as localized nodules at puncture sites, and surgical excision is often required.^2–7)^ Furthermore, the impact of immunomodulatory agents on the development or progression of CAG has not yet been fully elucidated, particularly in patients without a defined autoimmune disease.^6,7)^ This report describes a case in which a patient with an initially uneventful postoperative course following CAC developed severe CAG shortly after the initiation of dupilumab. The temporal association raises the possibility that immune modulation at the time of cyanoacrylate exposure may influence the severity or clinical course of granulomatous reactions, underscoring the need for careful patient assessment and long-term follow-up.

## Case Report

### Clinical course

A 74-year-old female (body mass index 19.1, Clinical–Etiologic–Anatomic–Pathophysiologic [CEAP] classification C2) with a history of chronic eczema who occasionally used antihistamines underwent bilateral great saphenous vein (GSV) CAC (VenaSeal; Minneapolis, MN, USA) under local and intravenous anesthesia. Sixteen centimeters of the left GSV and 22 cm of the right GSV were treated, using a total of 1.6 mL of cyanoacrylate. The procedure was uneventful, and she was discharged with compression therapy and analgesics. One week later, no complications were observed.

On postoperative day (POD) 15, dupilumab therapy was initiated by dermatologists at another hospital for refractory chronic eczema that had been inadequately controlled with antihistamines.

**Fig. 1:**
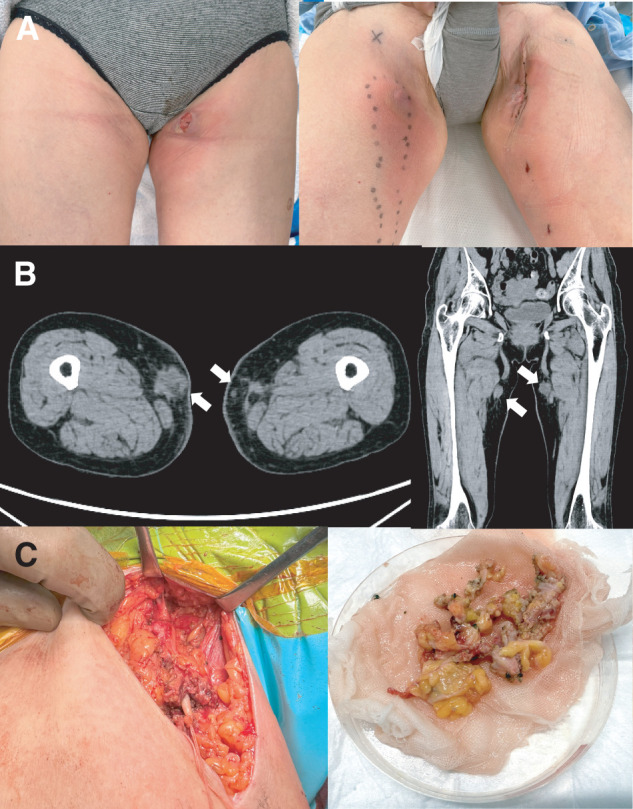
(**A**) Clinical findings. Left: POD 56 showing tender, erythematous nodules with partial skin ulceration in the left inguinal region prior to the first surgical excision. Right: POD 64, demonstrating postoperative wounds and residual inflammatory nodules in the right inguinal region following the second excision. (**B**) Plain CT demonstrating multiple subcutaneous nodules (arrows) along the bilateral great saphenous vein regions without evidence of abscess formation. (**C**) Intraoperative findings and resected specimens obtained on POD 93, showing cyanoacrylate granulomas excised from the disrupted great saphenous vein. CT: computed tomography, POD: postoperative day

On POD 16, the day after dupilumab administration, the patient developed fever, painful inguinal nodules, and erythema. Laboratory tests showed only mild elevations in both leukocyte count (10600/µL) and C-reactive protein (3.3 mg/dL). The leukocyte differential was as follows: neutrophils 84.6%, eosinophils 2.9%, basophils 0.5%, lymphocytes 6.3%, and monocytes 5.7%. For the severe allergy-like symptoms, a 3-day course of steroid pulse therapy was administered, along with antihistamines for 40 days. Following consultation with the dermatology team, dupilumab was discontinued immediately on POD 16, after the onset of systemic symptoms. Computed tomography demonstrated multiple subcutaneous nodules along the GSV regions without abscess formation (**Fig. 1B**). Despite prompt discontinuation of dupilumab, the inflammatory nodules continued to enlarge. By POD 56, the nodule in the left inguinal region had rapidly enlarged with partial skin ulceration, requiring surgical excision (**Fig. 1A**, left). By POD 64, nodules in the right inguinal region had also enlarged, and surgical excision was performed (**Fig. 1A**, right). Subsequently, by POD 93, painful and warm inflammatory nodules progressed along the medial thighs of both limbs, necessitating bilateral wide excision.

**Fig. 2:**
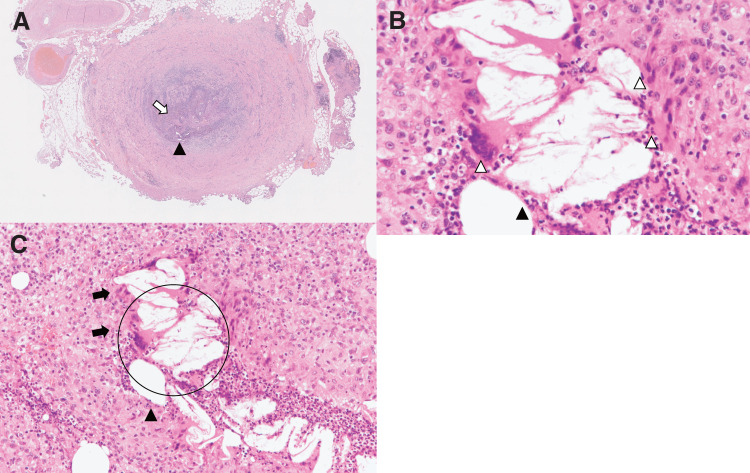
Histopathological findings of the excised venous nodule. Histopathological features of the venous nodule excised after a cyanoacrylate-induced foreign-body reaction. (**A**) Inflammatory cell infiltration is observed within the venous lumen (▲). Empty clefts resulting from dissolution of cyanoacrylate during tissue processing (▲) are present, surrounded by a histiocytic reaction consistent with a foreign-body response. (**B**) High-power view (×400) highlighting foreign-body–type multinucleated giant cells (∆), histiocytic aggregation surrounding cyanoacrylate-related voids (▲), and inflammatory infiltrates. (**C**) Another section of the same vessel (×200) demonstrates disruption of the venous wall (➡), extravascular inflammatory cell infiltration (circled areas), irregular voids suggestive of extravasated foreign material, and inflammatory infiltrates (▲).

Bacterial cultures of the excised specimens were negative. Histopathological examination confirmed a foreign-body–type granuloma (**Fig. 2A**). Details are described in the “Histopathological findings” section.

### Operative findings

The first surgical excision was performed on POD 56 under general anesthesia, targeting the nodules in the left inguinal region. Multiple small skin incisions were made along the course of the left GSV from the inguinal region to the above-knee level, and irregular subcutaneous nodules were excised as completely as possible.

Intraoperatively, the GSV was excised together with the surrounding inflammatory tissue. However, the venous structure was markedly disrupted and could not be clearly distinguished from the adjacent soft tissue. This was attributed to extravasation of cyanoacrylate beyond the venous wall, which had resulted in degeneration and inflammatory alteration of the surrounding tissue. Although the nodules were macroscopically located outside the expected venous lumen, approximately 2–3 cm away from the original GSV course, the boundary between cyanoacrylate-containing tissue and normal subcutaneous tissue was indistinct. Consequently, resection was necessarily limited to areas that appeared macroscopically involved.

On POD 64, a similar excision procedure was performed for the nodules in the right inguinal region, again including resection of the disrupted GSV together with the surrounding inflammatory tissue.

At the reoperation performed on POD 93, inflammatory changes had progressed longitudinally along the medial thighs of both limbs. Continuous skin incisions were made from the inguinal to the above-knee regions bilaterally, and wide excision was performed, including subcutaneous tissue containing cyanoacrylate deposits and inflammatory changes where the border with normal tissue was unclear (**Fig. 1C**). Complete en bloc removal of an anatomically intact cyanoacrylate-treated GSV was not feasible at this stage, because the venous structure had been extensively disrupted and could no longer be clearly identified intraoperatively. However, all identifiable cyanoacrylate-containing tissue and disrupted venous remnants were excised as completely as possible.

These operative findings indicate that cyanoacrylate extravasation can result in extensive inflammatory spread beyond the treated vein, necessitating a much wider dissection and excision than is typically anticipated in cases of cyanoacrylate-associated granuloma.

### Histopathological findings

Histopathological examination of the excised lesions revealed a foreign-body granulomatous reaction characterized by aggregates of histiocytes and foreign-body–type multinucleated giant cells surrounding cleft-like spaces, which were interpreted as voids left by cyanoacrylate that had dissolved during routine tissue processing (**Fig. 2A**). Inflammatory cell infiltration was observed within the venous lumen, predominantly composed of neutrophils and mononuclear cells, together with formation of foreign-body–type granulomas.

High-power examination demonstrated foreign-body–type multinucleated giant cells and histiocytic aggregation surrounding cyanoacrylate-related voids (**Fig. 2B**).

In additional sections from the treated GSV, disruption of the venous wall structure, extravascular extension of inflammatory infiltration, and extravasated foreign material were identified (**Fig. 2C**). Marked accumulation of histiocytes and neutrophils was present around the cleft-like spaces, indicating that the inflammatory process had extended beyond the venous wall into the surrounding soft tissue.

On hematoxylin–eosin staining, cyanoacrylate typically appears as a pale, amorphous gray-white substance with mild birefringence under polarized light. However, during routine paraffin embedding, the polymer frequently dissolves owing to alcohol and xylene processing, leaving empty clefts corresponding to the original location of the adhesive. The predominance of such voids rather than retained material in the present case is therefore considered to be attributable to processing-related dissolution. Chronic inflammatory cell infiltration, mainly composed of macrophages, epithelioid cells, and lymphocytes, is commonly observed in this setting, and the extent of the lesion varies depending on the amount of residual or extravasated adhesive and the host inflammatory response.^5)^

Histopathological examination of nodules located at a distance from the treated GSV revealed findings identical to those observed adjacent to the vein. These lesions consisted of foreign-body–type granulomatous inflammation with aggregates of histiocytes and multinucleated giant cells surrounding amorphous material or empty clefts consistent with dissolved cyanoacrylate. No lymph node architecture, including lymphoid follicles, germinal centers, or a preserved nodal capsule, was identified. Accordingly, these distant nodules were interpreted as cyanoacrylate-associated granulomatous lesions rather than lymph nodes.

### Review of the literature on CAG

Several cases of CAG after CAC have been reported in the literature. These cases are summarized in **Table 1**. As summarized in **Table 1**, the majority of previously reported cases of CAG developed several months after CAC and were typically localized. In contrast, the present case demonstrated an unusually early onset, occurring within weeks of treatment, which represents one of the earliest reported timings to date. Moreover, the continuous bilateral progression with longitudinal extension along both thighs, necessitating repeated surgical excisions, is exceedingly rare among reported cases. Notably, only a limited number of reports have addressed the potential influence of immune-modulating drugs on the development or progression of CAG, further underscoring the uniqueness and clinical relevance of the present case. Most granulomas developed between 3 and 12 months after treatment, typically at puncture sites or along the treated saphenous vein. Surgical excision was required in the majority of cases, whereas conservative management with antihistamines and other medications was rarely sufficient. Histopathological findings consistently revealed foreign-body granulomatous inflammation surrounding cyanoacrylate fragments. Although most cases presented with clinical symptoms requiring surgical excision, a few asymptomatic cases have also been identified on follow-up histological examination.^8,9)^

**Table 1:** Previously reported cases of CAG after CAC

Author (year)	Age (years)/sex	N	Product	Chief complaint	Location	Onset	Treatment	Outcome
Parsi et al.^8)^ (2020)	27.5/M:2	2	VenaBlock	NR	Right calf	12 months (histologic detection)	Observation	Asymptomatic
Almeida et al.^9)^ (2020)	49/F	1	VenaSeal	NR	Medial thigh along treated left GSV	5.5 years (histologic detection)	Observation	Asymptomatic
Zierau et al.^2)^ (2020)	NA	4	VenaSeal	NR	Access (puncture) site (per-case location NR)	NR (excision at 10–12 months post-CAC)	Surgical removal of a foreign body	No recurrence
Langridge et al.^3)^ (2020)	54/F	1	VenaSeal	Painless left anteromedial thigh lump	Left thigh, extravascular (GSV)	9 months post-CAC	Surgical removal of a foreign body	No recurrence
Sermsathanasawadi et al.^4)^ (2021)	74.3/F:3	3	VenaSeal	Puncture-site granuloma/abscess (GSV)	Access (puncture) site of the GSV; laterality: right 1/left 2 (per-case laterality not reported)	3–5 months post-CAC	Surgical removal of a foreign body	No recurrence
Athavale et al.^5)^ (2023)	82/M	1	VenaSeal	Enlarging painless subcutaneous lumps with erythema/drainage	Right GSV tract—proximal calf; later distal calf and groin	3 months post-CAC	Surgical removal of a foreign body	Healed after additional excision
Maraccaci et al.^7)^ (2025)	52/M	1	VenaSeal	Swelling/drainage and pain	Right lower leg	4 months post-CAC	Planned surgical removal of a foreign body	NR
Suzuki and Nakayama^6)^ (2025)	81/F	1	VenaSeal	Painful subcutaneous nodules	Left thigh, extravascular (GSV)	50 days post-CAC	Surgical removal of a foreign body	No recurrence

Adapted from previously published reports, including Parsi et al.^8)^ (2020), Zierau et al.^2)^ (2020), Almeida et al.^9)^ (2020), Langridge et al.^3)^ (2020), Sermsathanasawadi et al.^4)^ (2021), Athavale et al.^5)^ (2023), and Maraccaci et al.^7)^ (2025).

CAG: cyanoacrylate granuloma; CAC: cyanoacrylate closure; F: female; GSV: great saphenous vein; M: male; NR: not reported.

Compared with these previously published reports, our case was unique in demonstrating an unusually early onset, bilateral involvement, and progressive course requiring repeated surgical excision. In Japan, cases of CAG have been reported in patients with autoimmune diseases; however, immunomodulatory agents were not administered at the time of CAC in the reported cases. Therefore, a direct causal relationship between immunomodulatory therapy and granuloma formation cannot be established based on the existing literature. Rather, these observations suggest that the underlying immunological background, rather than autoimmune disease itself, may influence the host response to cyanoacrylate.^6)^

### CAG

Granuloma represents a localized foreign-body reaction characterized by aggregates of macrophages and multinucleated giant cells. CAG is considered a foreign-body–type granulomatous reaction to cyanoacrylate extravasated into perivenous tissue. It most commonly develops around puncture sites and usually appears several months after treatment, although delayed onset beyond 10 months has also been described.^2)^ The incidence of granuloma formation at the puncture site has been reported to be 2.3% in studies including only cases without catheter recapture at withdrawal, whereas the occurrence of granulomas along the treated vein has been reported to be 0.0041%.^1,4)^

Clinically, CAG presents as subcutaneous nodules with erythema, pain, or fever. Notably, long-term observational studies of treated veins for research purposes have demonstrated persistent inflammatory histopathological responses even several years after the procedure.^8,9)^ The pathogenesis is likely related to unresolved acute inflammation and subsequent chronic foreign-body reaction culminating in granuloma formation. Risk factors include inadvertent cyanoacrylate leakage into the subcutaneous tissue, residual glue at the puncture site, and premature catheter withdrawal before complete polymerization of the final injection. Failure to recapture the catheter tip before withdrawal has been significantly associated with increased risk.^4)^

Although conservative management with anti-inflammatory drugs, antihistamines, or short courses of corticosteroids may be attempted, such treatment is generally ineffective, and most patients ultimately require surgical excision of the foreign material. While CAG is usually sterile, secondary infection has occasionally been reported. Preventive strategies emphasize meticulous catheter technique to minimize subcutaneous leakage.^2,4)^

## Discussion

Most previously reported cases of CAG have arisen at puncture sites or as isolated subcutaneous nodules, and extensive or recurrent disease is uncommon.^2,4)^ Furthermore, CAG typically appears several months after CAC, with only rare exceptions. In contrast, the present case demonstrated an exceptionally rapid onset—emerging the day after the first dupilumab dose—and exhibited a bilateral, longitudinally progressive pattern that is rarely described in the literature. These features underscore the highly unusual nature of this presentation.

Dupilumab is a monoclonal antibody that targets the interleukin-4 receptor µ, inhibiting IL-4 and IL-13 signaling and thereby modulating the T-helper type 2 (Th2) cytokine pathway. Although not considered broadly immunosuppressive, dupilumab has been associated with granulomatous dermatitis and paradoxical inflammatory reactions, suggesting that it may alter host responses to foreign materials.^10)^ Recent immunologic insights support this hypothesis. Dupilumab may shift immune responses toward Th1/Th17 pathways, which are known to promote granulomatous inflammation.

Collectively, the unusually rapid onset, bilateral distribution, and progressive course requiring repeated excisions in our patient suggest that immune modulation at the time of cyanoacrylate exposure may have influenced the subsequent granulomatous response. This pattern has rarely been reported worldwide, making this case a meaningful contribution to the growing understanding of CAC–related foreign-body reactions in patients with altered immune backgrounds. Although perioperative use of such agents would typically be avoided, patients may receive biologics from other providers for dermatologic or allergic diseases, making prevention challenging.

This case highlights the importance of considering concomitant or recent biologic or immunomodulatory therapy, as well as the timing of such treatments, as potential modifiers of foreign-body reactions following CAC. This case suggests the importance of careful follow-up in patients receiving biologics.

## Conclusion

We reported a rare case of severe CAG occurring shortly after CAC in a patient receiving dupilumab. Notably, disease progression continued despite early discontinuation of dupilumab, suggesting that immune modulation at the time of cyanoacrylate exposure may have initiated a sustained granulomatous response. Granulomatous reactions following CAC may be influenced by immune modulation or altered immunological background, rather than by autoimmune disease itself. Careful perioperative assessment and follow-up are warranted in patients receiving biologic agents. In addition, further accumulation of cases is required to clarify its pathophysiology and establish appropriate management strategies.
